# Bioinspired Immobilization of Glycerol Dehydrogenase by Metal Ion-Chelated Polyethyleneimines as Artificial Polypeptides

**DOI:** 10.1038/srep24163

**Published:** 2016-04-07

**Authors:** Yonghui Zhang, Hong Ren, Yali Wang, Kainan Chen, Baishan Fang, Shizhen Wang

**Affiliations:** 1 Department of Chemical and Biochemical Engineering, College of Chemistry and Chemical Engineering, Xiamen University, Xiamen, 361005, China; 2 The Key Lab for Synthetic Biotechnology of Xiamen City, Xiamen University, Xiamen, Fujian, 361005, P. R. China

## Abstract

In this study, a novel, simple and generally applicable strategy for multimeric oxidoreductase immobilization with multi-levels interactions was developed and involved activity and stability enhancements. Linear polyethyleneimines (PEIs) are flexible cationic polymers with molecular weights that span a wide range and are suitable biomimic polypeptides for biocompatible frameworks for enzyme immobilization. Metal ion-chelated linear PEIs were applied as a heterofunctional framework for glycerol dehydrogenase (GDH) immobilization by hydrogen bonds, electrostatic forces and coordination bonds interactions. Nanoparticles with diameters from 250–650 nm were prepared that exhibited a 1.4-fold enhancement catalytic efficiency. Importantly, the half-life of the immobilized GDH was enhanced by 5.6-folds in aqueous phase at 85 °C. A mechanistic illustration of the formation of multi-level interactions in the PEI-metal-GDH complex was proposed based on morphological and functional studies of the immobilized enzyme. This generally applicable strategy offers a potential technique for multimeric enzyme immobilization with the advantages of low cost, easy operation, high activity reservation and high stability.

Natural multi-enzyme complexes are biomolecular machines that are assemblies of several enzymes and have been perfected over millions of years of evolution. Various synergistic interactions induce the formation of precise quaternary structures that guarantees high catalytic efficiency and stability^1^. The high affinities of metal ions for protein side chain provide considerable interactions and folding energies compared with weak interactions, i.e., hydrophobic interaction and hydrogen bonds. The metal ions that are in natural protein-protein interfaces stabilize quaternary or even the supramolecular protein structures and mediate transient protein-protein interactions. Additionally, both the structural and functional contributions of a metal ion are responses to the factors that affect the coordination of the metal ion, e.g., the presence of the metal ion itself, external chelators and pH value^2^. Inspired by their natural functions in coordination, metal ions are increasingly being applied to the assembly of protein architecture^3^.

Linear polyethyleneimines (PEIs) are flexible cationic polymers with molecular weights that vary over a wide range and are suitable for mimicking polypeptides. Linear PEIs contain uniform secondary amino groups that simplify the mechanistic study of the interactions between PEIs and enzymes, whereas branched PEIs contain primary, secondary and tertiary amino groups. Specifically, PEIs are biocompatible and are widely used as efficient gene carriers^4^. A previous study indicated that coating surfaces with hydrophobic polycations such as N,N-dodecyl, methyl-PEI kill viruses on-contact *in vitro* while being physically safe for human beings^5^. The use of PEIs has been reported in enzyme immobilization, for example, in the coating of enzyme surfaces^6^,^7^,^8^, PEI-based cross-linking to form PEI-CLEAs^9^ and as promoters for enzyme immobilization^10^. However, the majority of reports have used branched PEIs and merely regarded them as modifiers of solid carriers.

Oxidoreductases are playing an increasingly important role in the diverse productions of fine, special, and bulk chemicals due to their exceptional selectivity^11^. Most oxidoreductases are multimeric enzymes that are more fragile than hydrolases and do not survive the challenges of extreme temperature, pH, co-solvent, and shear and surface forces when applied in industrial biocatalysis processes. The stabilities of oxidoreductases are frequently fundamental limitations of their applications. Inactivation typically begins with the loss of the integrity of the quaternary structure of the multimeric oxidoreductases followed by the loss of the tertiary structure (for the subunits of enzymes) and then an irreversible denaturation step^12^,^13^. The stability of oxidoreductases can be improved by immobilization^14^, medium engineering^15^ or protein engineering^16^,^17^. Typical immobilization strategies include covalent immobilization, entrapment and adsorption^18^,^19^. However, due to distortion of the tertiary structure, blockage of the active site, and diffusion limitions^20^, approaches that can both maintain high enzyme activity and systematically improve enzyme stability are lacking.

In this paper, the potential of metal ion-chelated linear PEIs applied as artificial polypeptides for bioinspired immobilization of multimeric oxidoreductases was studied. Glycerol dehydrogenase (GDH) from *Klebsiella pneumonia* was selected as a model of multimeric dehydrogenases. The heterofunctional interactions of metal ion-chelated linear PEIs with amino acid residues, including hydrogen bonds, electrostatic forces and coordination bonds, were considered. Morphological and functional studies of the enzyme immobilization by the metal-ion coordinated PEIs were performed to verify the mechanism of the improvements in enzyme activity and stability.

## Results

### Effect of Metal ion and PEI on enzyme activity

**Figure 1:**
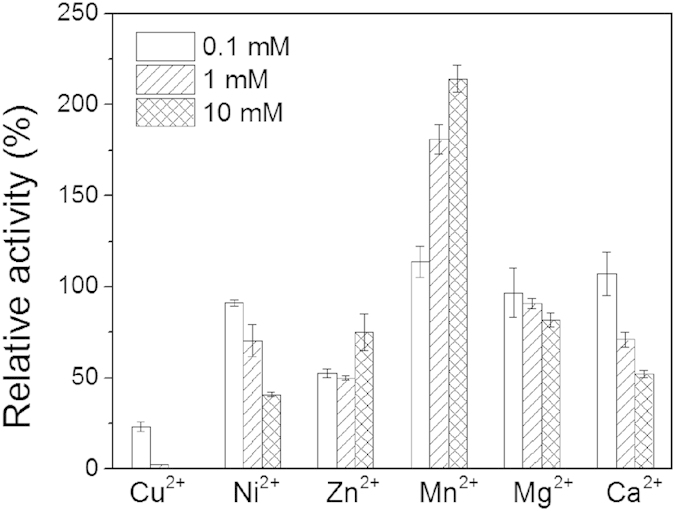
The effect of metal ions on GDH activity. Reaction conditions: glycerol, 0.2 M; NAD^+^, 2 mM; enzyme, 0.25 μM; pH, 10.0; temperature, 30 °C. The relative activity is expressed as a percentage of the original activity assayed without the metal ions. The activity was assayed as described in the Methods section after incubating GDH with the various metal ions for 30 min.

To obtain immobilized enzymes with preferable activities, it was necessary to evaluate the effects of the metal ions on the GDH activity according to the selected metal ion. Metal ions not only act as coordination cross-linkers between linear PEIs and enzymes but can also be activators or inhibitors of enzymes in the immobilization process due to metal-ion coordinated PEIs. Structure study of GDH from *Bacillus stearothermophilus* demonstrated that divalent metal ion, Zn^2+^ was required for the multimer formation and enzymatic catalysis of GDH^21^. The natural GDH-bound Zn^2+^ were substituted with several divalent metal ions and the enzyme activity was significantly altered^22^,^23^. Therefore we selected six divalent metal ions (Cu^2+^, Ni^2+^, Zn^2+^, Mn^2+^, Mg^2+^ and Ca^2+^) to investigate the effects of metal ions on GDH activity. The GDH activities were assayed after incubating GDH with metal ions for 1 h at 4 °C. Figure 1 demonstrates that Mn^2+^ improved the GDH activity by 1.1-fold, whereas the other five metal ions inhibited the enzyme to varying degrees at high concentrations. Therefore, these properties made Mn^2+^ an outstanding metal ion for coordinated immobilization.

**Figure 2:**
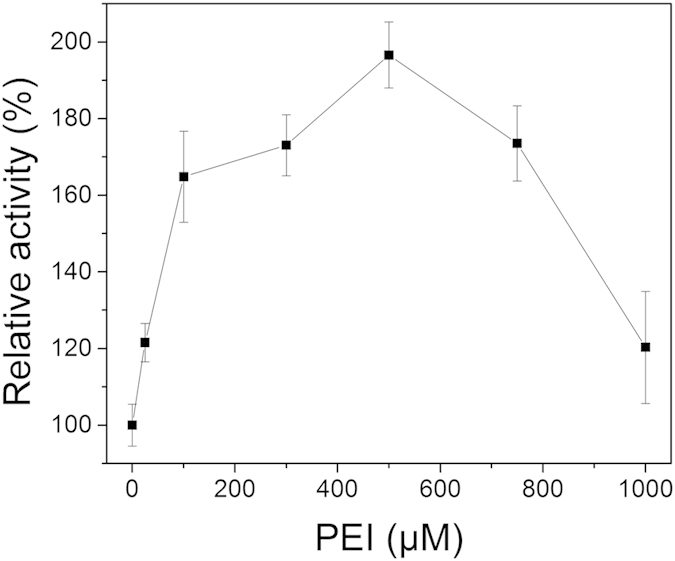
The effect of PEI concentration on GDH activity. The GDH activity was assayed as described in the Methods section after incubating GDH with linear PEI at concentrations ranging from 0 to 500 μM for 1 h. Reaction conditions: glycerol, 0.2 M; NAD^+^, 2 mM; enzyme, 0.25 μM; pH, 10.0; temperature, 30 °C. The relative activity was calculated as the percentage of the original activity assayed before incubation with linear PEI.

The effects of PEI concentration on GDH activity were tested by assaying the relative activities after the incubation of GDH (0.5 mM) with linear PEI at concentrations ranging from 0 to 500 μM for 1 h. As shown in Fig. 2, increasing the PEI concentration led to increases in the immobilized GDH activity. At the final PEI concentrations of 100 μM and 500 μM, the GDH activities were increased by 58.8% and 90.6%, respectively.

### Coordination immobilization of GDH

**Figure 3:**
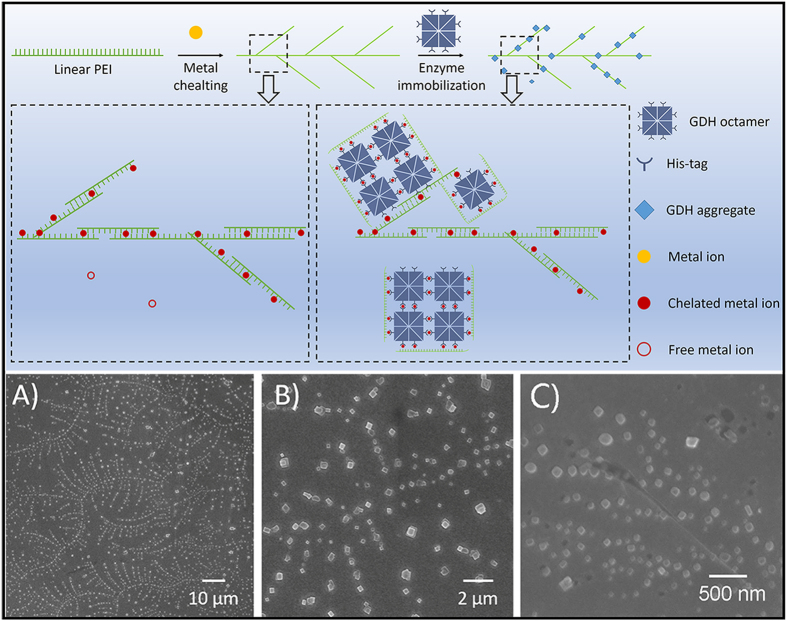
(Top) Illustration of the formation of the PEI-metal-GDH by multi-level. (Bottom) SEM image of (**A**) Overall pattern of PEI-Mn^2+^-GDH. (**B**) The GDH-metal assemblies via metal coordination interaction slice (**C**) Free GDH octamer. The PEI-Mn^2+^-GDH complex was prepared as descripted in Method section.

Linear PEIs acted as framework for GDH immobilization and provided the multi-amine group for coordination with the metal ion. Scanning electron microscopy (SEM) images of GDH immobilized with manganese-chelated PEIs are presented in Fig. 3. The PEI-Mn^2+^-GDH formed a branched feather-like structure (Fig. 3A). Dynamic light scattering analysis of the PEI-Mn^2+^-GDH (Fig. S1) indicated that the diameters of the immobilized enzymes were in the range of 100 to 1000 nm, and the majority of the enzymes had diameters of 250–650 nm. The process of the promotion of GDH immobilization by the metal-ion coordinated PEI is illustrated in Fig. 3(Top). First, linear PEIs attached together to form a large web structure through metal coordination binding. As shown in Fig. 3A, the obtained PEI web formed a long PEI string with a length over 200 μm, suggesting that linear PEIs was able to sufficiently expand probably due to the internal electrostatic repulsions between the amine groups of the PEIs at neutral pH^24^. After the PEI framework was formed, GDH was added and linked to the manganese-chelated PEIs. Meanwhile, the GDH monomers were able to couple with other GDH monomers^25^. Homologous oligomerization of GDH resulted in an octamer (50–100 nm in edge length, Fig. 3C) that could be further assembled into a larger inerratic enzyme-metal slice (250–650 nm in edge length, Fig. 3B) via the metal coordination interaction. Additionally, the oligomeric GDH assemblies were coated and stabilized by the excessive manganese-chelated PEIs, which further prevented the disassociation of the GDH subunits. Metal-mediated oligomeric assemblies of enzymes have been reported to improve the crystallizability of proteins and promote protein crystallization by enhancing the interactions of the subunits of the enzyme^26^,^27^,^28^,^29^, which may explain the formation of the inerratic enzyme-metal slice.

### Analysis of the interaction of PEI-Mn^2+^ and GDH

**Figure 4:**
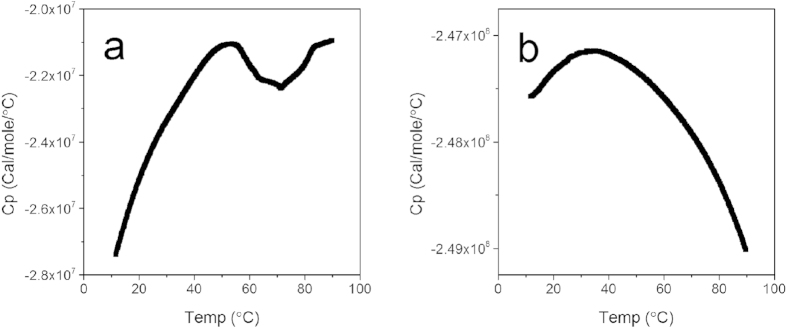
Differential scanning calorimetric curves of free GDH (**a**) and immobilized GDH (**b**).

The interaction of PEI-Mn^2+^ and GDH was analyzed with differential scanning calorimetry (DSC). To characterize the denaturation behavior of GDH and PEI-Mn^2+^-GDH, the temperature dependence of the differential heat was measured. Moreover, the thermostability of PEI-Mn^2+^-GDH compared with that of free GDH was studied with an assay that measures the changes in the heat capacity of a given protein in solution during the thermal unfolding process. The heat capacity changes (ΔC_p_) of free GDH (Fig. 4a) and PEI-Mn^2+^-GDH (Fig. 4b) were tested. The ΔC_p_ typically ascribed to a hydrophobic effect is negative, which indicates that when the ordered waters from a hydrophobic surface move to the bulk solution, additional energy must be absorbed. The ΔC_p_ of PEI-Mn^2+^-GDH was one order of magnitude higher than that of free GDH, indicating that the immobilization greatly improved stability.

### Improvement of pH adaptability by coordination immobilization

**Figure 5:**
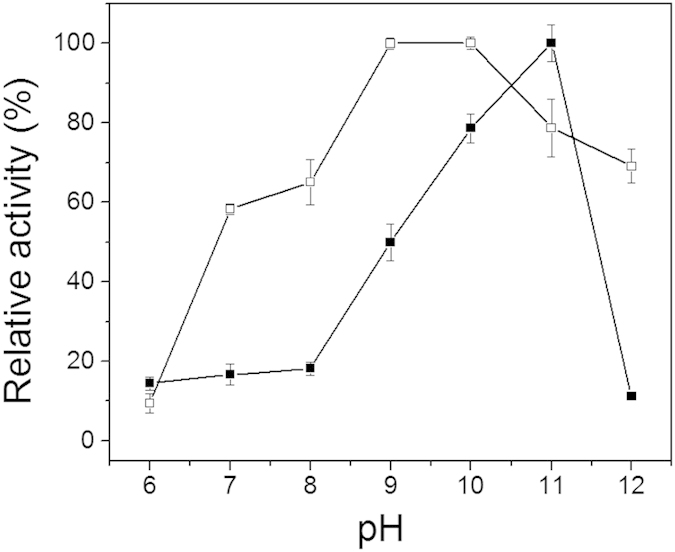
The effect of pH on the activities of free GDH and PEI-Mn^2+^-GDH (▪: free GDH; ◻: PEI-Mn^2+^-GDH). 100 mM sodium phosphate buffer (pH 6–8) and 1 M NH_4_Cl-NH_3_•H_2_O buffer (pH 9–12) were used to determine the optimal pH range. Reaction conditions: glycerol, 0.2 M; NAD^+^, 2 mM; enzyme, 0.25 μM; pH, 6–12; temperature, 30 °C. The relative activity at the optimum pH for free GDH were taken as 100%.

The optimal reaction pH for free GDH and PEI-Mn^2+^-GDH were determined. As shown in Fig. 5, the immobilized GDH changed the optimal pH from 11 to 10 and exhibited high activity in a relatively wide range of pH from 7 to 12, while free GDH only achieved high activity at pH 11. PEI-Mn^2+^-GDH exhibited a 2.9-fold increase in activity compared with free GDH. Immobilization greatly broadened the pH adaptability of GDH from strong alkaline into alkalescence, which will promote the application of GDH, particularly for coupling with other dehydrogenases for cofactor regeneration.

### Improvement of thermostability by coordination immobilization

**Figure 6:**
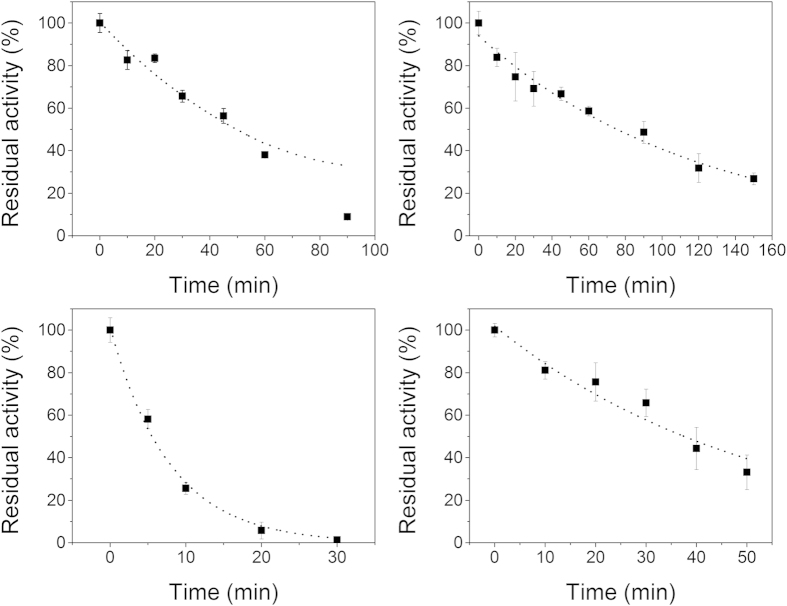
Thermostabilities of free GDH and immobilized GDH (a: free GDH at 70 °C; b: PEI-Mn^2+^-GDH at 70 °C; c: free GDH at 85 °C; d: PEI-Mn^2+^-GDH at 85 °C). The relative activity is expressed as a percentage of the original activity as assayed before incubation at the different temperature. Reaction conditions: glycerol, 0.2 M; NAD^+^, 2 mM; enzyme, 0.25 μM; pH, 10.0; temperature, 30 °C.

The thermostabilities of PEI-Mn^2+^-GDH and free GDH were studied at 70 °C and 85 °C. As shown in Fig. 6, PEI-Mn^2+^-GDH exhibited greater heat resistance than free GDH. After incubation for 90 min at 70 °C, PEI-Mn^2+^-GDH still presented nearly 50% of its initial activity, while free GDH was almost totally inactivated. Significant improvements in thermostability were also demonstrated at 85 °C; after incubation at this temperature for 30 min, free GDH was completely deactivated, but PEI-Mn^2+^-GDH only lost 35% of its initial activity.

The thermal deactivation kinetics of free and immobilized GDH were investigated and found to be first-order processes (Fig. 6). Therefore, the thermal deactivation kinetics were studied according to Eq.(1), where K_d_ and E_0_ are the deactivation rate constant and initial enzyme activity (E_0_ = 100%), respectively^30^,^31^.


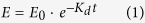


The deactivation rate constants and half-lives were calculated based on a first-order deactivation process (Table 1). The deactivation energies of free GDH and PEI-Mn^2+^-GDH were calculated according to Eq. 2.


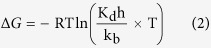


ΔΔG was defined as the deactivation energies of PEI-Mn^2+^-GDH minus the deactivation energies of free GDH. The ΔΔG values shown in Table 2 indicate that the immobilization increased the deactivation energy and improved the thermostability, especially at the high temperature.

### Kinetic study of the immobilized enzyme

The kinetic parameters of the free GDH and PEI-Mn^2+^-GDH in glycerol oxidation were determined using the Lineweaver-Burke method (Fig. S2). The Michaelis-Menten constant *K*_m_ increased slightly by 20%, which might have been due to the increased mass-transfer limitation^32^,^33^. Moreover, the catalytic number (*k*_cat_) and the catalytic efficiency (*k*_cat_/*K*_m_) of PEI-Mn^2+^-GDH were 1.7- and 1.4-fold those of the free enzyme (Table 3).

### Reusability of PEI-Mn^2+^-GDH nanoparticles

The reusability of PEI-Mn^2+^-GDH nanoparticles was investigated. Fig. S3 shows that the activity of PEI-Mn^2+^-GDH gradually decreased over 5 cycles. PEI-Mn^2+^-GDH retained 71% and 53% of its initial activity after cycling through 3 and 5 successive reactions, respectively. The decrease in the activity of the recycled catalyst may be due to the leakage of GDH. The coordination interaction between the enzyme and the PEI carrier that was cross-linked by metal ions was influenced by pH and ion strength and was a relatively weak bond compared with a covalent bond. The flexibility of the nanoparticles provides the possibilities of reversible immobilization and the enhancement of immobilization strength by condition optimization and process operations.

## Discussion

The majority of oxidoreductases denature quickly at high temperatures, which greatly limits their applications. There has been reports on thermophilic dehydrogenase in the aqueous phase or specific dehydrogenases in organic phase catalysis^34^,^35^. However, those thermostable dehydrogenases require an intrinsically thermophilic nature or organic solvent tolerance, which are usually largely dependent on enzyme molecular modification or the discovery of extremozymes. To engineer oxidase-reductase from biological entities into industrial applicable catalyst, some old immobilization techniques based on covalent bonding or absorption have been widely used to improve enzyme catalytic properties like activity and stability and permit catalyst reuse. However, it often resulted in generating steric hindrances, making serious impairment to enzyme activity.

Recent studies about GDH immobilization have demonstrated that traditional immobilization approaches via absorption and covalent bonds could cause severely enzyme activity lose. Kumar *et al*. reported that GDH only retained 20% specific activity when the enzyme was absorbed with magnetically-separable mesoporous silica, and retained 27% specific activity when further crosslinking with glutaraldehyde treatment was implemented^36^. Agarose beads activated with different reactive groups including CNBr group, glyoxyl group and amine groups were used to covalently immobilize GDH and only retained 40–50% of enzyme activity in the present of polyethylene glycol (PEG)^37^. Activated silica coated magnetic Fe_3_O_4_ nanoparticles was used for the immobxilization of GDH via a glutaraldehyde linkage and resulted in 31% decrease of k_cat_ compared with that of free enzyme^38^. In our study, via noncovalent immobilization, PEI-Mn^2+^-GDH immobilization system was constructed and exhibited excellent activity and stability enhancement.

As the support and cross linker of the immobilized structure, PEI and Mn^2+^ manifested positive effect on GDH activity. Positive effects of Mn^2+^ ions on GDH activity have been reported^39^,^40^. Mn^2+^ is a Lewis acid and an electrostatic stabilizer with intermediate properties relative to these other ions, including its radius length and borderline hard-soft character. Mn^2+^ ions exhibit good electron delocalization and a high chelating ability^41^, which aid the formation of flexible coordination bonds^42^. Furthermore, affinity binding and the formation of manganese clusters have been reported to be responsible for coupling protons and transferring electrons in oxidation reactions^43^. Manganese can be replaced with other metals, i.e., magnesium and zinc^44^. Mn^2+^ has also been reported to be the activator of several oxidoreductases, including laccase, peroxidase, formate dehydrogenase and carbonyl reductase^45^,^46^,^47^,^48^. As the natural enzyme-bound metal ion, the increase of Zn^2+^ ion seem to had positive effect which was similar to Mn^2+^ and different from Cu^2+^, Ni^2+^, Mg^2+^ and Ca^2+^. The tendency difference between Zn^2+^ and other metal ion may be due to the different structural compatibility of metal ion to bind with GDH. 6× His tag was usually applied for recombinant protein purification by metal affinity chromatography. GDH was expressed with 6× His tag tail which could provide stable binding force with Mn^2+^ ion in our immobilization system. For enzyme without 6×His tag, there is still great chance to successfully immobilized using metal coordinated PEI if there are amide or imidazole ligands on the solvent accessible area^49^. For example, several enzyme-metal nanoflowers was successfully constructed without 6× His tag including albumin from bovine serum (BSA), α-lactalbumin, bovine milk, laccase, Glucose oxidase (GOx), horseradish peroxidase (HRP), and β-glucose^25^,^30^. Noteworthy, the utilization of this strategy in specific enzyme immobilization required careful studies and the obtained results could be varied case by case due to the difference in enzyme feature.

PEI is a strong polycation with a high proportion of amine protonation, which results in electrostatic attractions between the enzyme and PEI. Branched PEI has been reported to improve the activities of glucose dehydrogenase and lecitase^50^,^51^. The multimer form of GDH was well stabilized by the hydrogen bonds between PEI and the amino acid residues on the enzyme surface, which formed a very efficient macromolecular cage around the enzymes. The macromolecular cage prevents the dissociation of the subunits of multimeric enzymes which would cause enzyme inactivation, and results in better enzymatic performance^10^. Furthermore, repulsive forces between neighboring PEI chains on the surface were formed by the protonation of a high proportion of the amino groups, which resulted in an extended conformation that maximized the interaction with the enzymes.

Changes in pH affect the multiple interactions between GDH subunits, including hydrogen bonds, electrostatic interactions and salt bridges, which sustain the multimeric form of GDH and may lead to the dissociation of subunits and inactivation^52^. The metal ions formed coordination bonds with both PEI and the enzyme that acted as a gentle cross-linker that greatly inhibited the dissociation of the subunits. Additionally, the distinctive buffering capacity of linear PEI due to the large amount of secondary amine groups enhanced the resistance of the immobilized enzyme to pH variations.

Investigation of thermodynamic parameters such as changes in the Gibbs free energy (∆G) is good way for understanding the thermostability of biomolecules, providing further proof of thermostability enhancement by immobilization treatment. The increase of ΔG indicated that the metal-coordinated interaction of the subunits and the interaction between PEI and the enzyme reinforced the stability of the multimeric structure and prevented subunit dissociation and thus improved the thermostability. These properties could greatly expand the applications of GDH in multi-enzyme catalysis and biosensors^53^.

The kinetic study showed an enhancement in *k*_cat_ and *k*_cat_/*K*_m_, which can be primarily attributed to the stabilization of the multimeric structure and the activation effect of the metal ions on the enzyme activity. It has been reported that metal ions in immobilized nanostructures can act as activation factors as free metal ions in solution^25^. The immobilization of dehydrogenases typically decreases enzyme activity due to distortions of the tertiary structure, blocking of the active site, diffusion limitations caused by support and unsatisfactory enzyme aggregation^11^. Generally speaking, the loss of activity tends to be “compensated” for with other enzyme properties, such as thermostability^35^, operational stability, tolerance to organic solvents and reusability. Surprisingly, both activity enhancement and stability improvement were achieved by metal ion-coordinated PEI immobilization. First, the coordinate bonds formed by metal ion coordination between the imine group of the linear PEI and the enzyme amino acid residues were weaker than covalent bonds, which provided excellent flexibility to prevent the distortion of the enzyme structure relative to the use of chemical cross-linkers, i.e., glutaraldehyde. Reversible immobilization can be achieved to remove metal ions by chelation with chelating agents, such as EDTA. Second, for those enzymes that can be greatly activated by specific metal ions, the utilization of this specific metal ion as a cross-linker benefits the maintenance or even enhances enzyme activity. Third, immobilization using the metal-coordinated PEI enzyme generated nanoparticles with relatively small sizes of approximately 250–650 nm (Fig. 3B) and well-distributed metal-chelated PEI skeletons. The formation of nanoparticles was anticipated to reduce the negative effect of immobilization on the substrate/product diffusion. In contrast, the traditional CLEAs generated a massive enzyme aggregation at the micrometer or millimeter scale, which caused severe diffusion limitations^54^.

To sum up, a generally applicable method for the noncovalently immobilization of enzymes using metal-chelated linear PEI as a flexible framework was developed. This method greatly improved dehydrogenase stability. Linear polyethyleneimines are flexible cationic polymers with molecular weights that vary over a wide range that are suitable as biomimic polypeptides for the formation of biocompatible frameworks for enzyme immobilization. Metal ion-chelated linear PEIs exhibit heterofunctional interactions with amino acid residues that include hydrogen bonds, electrostatic forces and coordination bonds. Coordination bonds induced by metal ions endow structure, chemical reactivity and stimuli-responsiveness to enzymes and can also tune enzyme activity^55^. The results indicated that the proposed bioinspired immobilization strategy provided a novel technique for multimeric enzyme immobilization for biocatalysis. Additionally, due to the biocompatibility, oxidoreductases immobilized by metal ion-chelated linear PEIs have great potential applications as biosensors and *in vitro* diagnostic reagents^56^,^57^,^58^.

## Methods

### Materials

Glycerol, NAD^+^, poly (2-ethyl-2-oxazoline) (500kDa), CuSO_4_, NiSO_4_, ZnSO_4_, MnSO_4_, MgSO_4_ and CaCl_2_ were purchased from Sigma-Aldrich (Tianjin, China). Ampicillin and isopropyl-β-d-thiogalactoside (IPTG) were purchased from TransGen Biotech (Beijing, China). All other chemicals were of analytical grade and were purchased from Sangon Biotech (Shanghai, China). *E. coli* BL21(DE3) pET-32a-GDH was constructed in our laboratory (not published, detailed information was listed in Supplementary information).

### Expression and preparation of GDH

*E. coli* BL21(DE3) pET-32a-GDH was grown at 37 °C in 1 L shaking flasks containing 200 mL of LB (Miller) media with 100 mg/L ampicillin. The expression of recombinant GDH was induced by the addition of 1 mM IPTG at an optical density (OD_600_) of approximately 0.5 for 6 h at 30 °C. The cells were harvested by centrifugation at 10000 × g for 10 min at 4 °C and disrupted by ultrasonication. The cell debris was removed by 20 min centrifugation at 12000 × g at 4 °C.

Crude cell extract was then applied to a Histrap column (5 mL, (GE Healthcare Corp., Piscataway, NJ, USA)) equilibrated with bind buffer (20 mM sodium phosphate, 0.5 M NaCl, 20 mM imidazole, pH 7.4). The column was equilibrated with binding buffer and eluted with elution buffer (20 mM sodium phosphate, 0.5 M NaCl, 500 mM imidazole, pH 7.4) at a gradient concentration. The fractions with the desire activity were desalted and concentrated using a Macrosep Advance Centrifugal Device (cut-off 10 kDa, Pall, East Hills, NY, USA).

### GDH activity assay

The measurements of GDH activity were performed with testing of NADH (ε = 6.22/mM/cm) concentrations of 340 nm at 30 °C using a Thermo Scientific Multiskan Ascent Microplate Reader (Theromo Labsystems, Helsinki, Finland). The assay mixture contained 0.2 M glycerol, 2 mM NAD^+^, 1 M NH_4_Cl-NH_3 _H_2_O (pH 10.0) and 20 μL of enzyme solution. The volume of the reaction mixture was 200 μL in all cases. The reactions were initiated by the addition of the enzyme solutions. The enzyme activities were determined in triplicate. One unit of GDH activity was defined as the amount of enzyme required to catalyze the reduction of 1 μM NAD^+^ per minute.

### Preparation of linear PEI

Linear PEI was prepared using the protocol reported previously^59^. A mixture of 10 g (500 kDa) poly (2-ethyl-2-oxazoline) and 500 mL (24%) HCl in pear-shaped flask was refluxed for 108 h with vigorous stirring. Using an ice water bath, KOH was slowly added in portions to neutralize the acid and attain a pH between 8 and 9. The produced linear PEI (217 kDa) was filtered and dried under vacuum at −48 °C.

### Formation of the PEI-metal-GDH complex

Enzyme immobilization with metal-ion coordinated PEI was developed using metal ions as cross-linkers between the amine group of the PEI and the amino acid residues on the enzyme surface. A typical PEI-metal-GDH complex preparation was performed as follows.

Metal ion solution (0.2 mL, 1 M, final concentration = 9.9 mM) was added to 10 mL of linear PEI (1 mM, dissolved in distilled water, final concentration = 495 μM), vortexed for 30 s, and incubated for 10 min at 4 °C to obtain metal-chelated PEI. 10 mL of free GDH (50 μM, dissolved in 100 mM sodium phosphate buffer, pH 7.4, final concentration = 24.75 μM) was then mixed with metal-chelated PEI, vortexed for 30 s, and incubated at 4 °C for 30 min. The immobilized GDH was collected by centrifugation (1000 × g for 5 min at 4 °C). The precipitate was then washed three times with sodium phosphate buffer (100 mM, pH 7.4) to remove the unbound components and stored at 4 °C. The morphology of the immobilized enzyme was characterized by scanning electron microscopy (SEM) and dynamic light scattering (DLS). For the SEM, 2 μl of immobilized enzyme was dropped on a silicon grid and allowed to evaporate overnight, and the sample was then coated with platinum (2-nm thickness using a JEOL JFC 1600 (JEOL, Tokyo, Japan) with an electric current of 10 mA for 30 s before imaging with a Zeiss Sigma SEM (Carl-Zeiss AG, Germany). For the DLS, the measurements were performed on a Malvern Nano ZS (Malvern Instruments, Worcestershire, UK).

### The reuse of the PEI-Mn^2+^-GDH

The reusability of the PEI-Mn^2+^-GDH was studied by recycling the immobilized enzyme through centrifugation (1000 × g for 5 min at 4 °C) after the first GDH activity assay followed by three washes with sodium phosphate buffer (100 mM, pH 7.4). The activity of the recycled biocatalyst was tested. The activity during the first run was defined as 100%, and those during the successive runs are presented as relative activities.

### Differential scanning calorimetry measurements

The differential scanning calorimetry measurements were performed on a MicroCal VP-DSC system (GE Healthcare, Northampton, Massachusetts). All samples with enzyme concentrations of 25 μM were degassed and measured at a scan rate of 60 °C/h with scan temperatures from 10 °C to 90 °C. Data analysis was performed using Origin (version 7.0) software provided by MicroCal.

## Additional Information

**How to cite this article**: Zhang, Y. *et al*. Bioinspired Immobilization of Glycerol Dehydrogenase by Metal Ion-Chelated Polyethyleneimines as Artificial Polypeptides. *Sci. Rep.* **6**, 24163; doi: 10.1038/srep24163 (2016).

## Supplementary Material

Supplementary Information

## Figures and Tables

**Table 1:** Deactivation rate constants and half-lives of free GDH and immobilized GDH.

Temperature (^o^C)	GDH		PEI-Mn^2+^-GDH	
	K_d_ (min^−1^)	t_1/2_ (min)	K_d_ (min^−1^)	t_1/2_(min)
70	0.014 ± 0.001	49.5	0.008 ± 0.001	86.6
85	0.127 ± 0.009	5.5	0.019 ± 0.002	36.5

**Table 2:** Thermodynamic parameters of deactivation for the free and immobilized GDH.

Temperature(^o^C)	ΔG of free GDH	ΔG of PEI-Mn-GDH	ΔΔG
	(KJ/mol)	(KJ/mol)	(KJ/mol)
70	47.18	48.87	1.69
85	42.07	48.05	5.98

Reaction conditions: glycerol, 0.2 M; NAD^+^, 2.0 mM; enzyme, 0.25 μM; pH, 10.0; temperature 70 °C.

**Table 3:** Kinetic parameters of free GDH and immobilized GDH.

GDH	*K*_m_ (mM)	*V*_m_ (U/mg)	*k*_cat_ (s^−1^)	*k*_cat_/*K*_m_ (×10^3^M^−1^s^−1^)
Free	21.6 ± 2.2	2.26 ± 0.04	2.03 ± 0.03	0.094 ± 0.004
PEI-Mn^2+^-GDH	26.0 ± 1.4	3.73 ± 0.04	3.36 ± 0.03	0.129 ± 0.002

The GDH activity was determined by the oxidation of glycerol (25–200 mM) in 1 M NH_4_Cl-NH_3_ H_2_O (pH 10.0) at 30 °C using 0.25 μM GDH and 2 mM NAD^+^.
