# Supplementary material for: Bioinspired Immobilization of Glycerol Dehydrogenase by Metal Ion-Chelated Polyethyleneimines as Artificial Polypeptides
Source: Sci Rep. 2016 Apr 7;6:24163. doi: 10.1038/srep24163 (PMC4823755; doi:10.1038/srep24163)
Supplement: Supplementary Information [file srep24163-s1.pdf]

Supplementary Information

Bioinspired Immobilization of Glycerol Dehydrogenase by Metal Ion-Chelated Polyethyleneimines as Artificial Polypeptides

Yonghui Zhang[1], *Hong Ren*[1], *Kainan Chen*[1], *Baishan Fang*[1,2]*, *Shizhen Wang*[1,2]*

**Co-corresponding author*

[1]Department of Chemical and Biochemical Engineering, College of Chemistry and Chemical Engineering, Xiamen University, Xiamen, 361005, China. [2]The Key Lab for Synthetic Biotechnology of Xiamen City, Xiamen University, Xiamen, Fujian, 361005, P. R. China.

Correspondence and requests for materials should be addressed to B. F. (email: fbs@xmu.edu.cn) and S.W. (email: szwang@xmu.edu.cn).

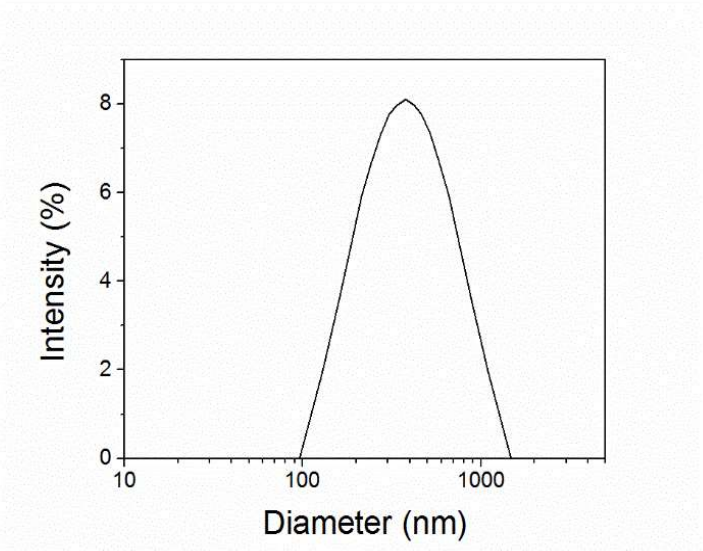

Figure S1. Dynamic light scattering analysis of PEI-Mn^{2+}-GDH

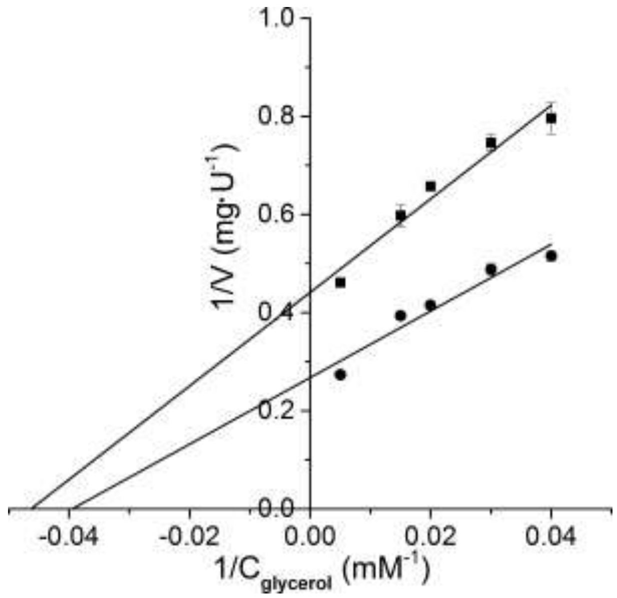

Figure S2. Double reciprocal plots of initial reaction rates for variable concentrations of glycerol (25 – 200 mM) of PEI-Mn2^{+}-GDH (■) and free GDH (●).

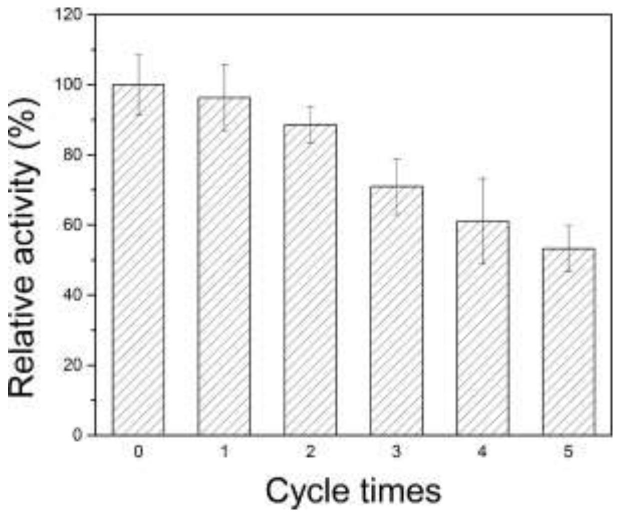

Figure S3. Reusability of PEI-Mn^{2+}-GDH. The relative activities are expressed as percentages of the original activity. Reaction conditions: glycerol, 0.2 M; NAD^+, 2mM; enzyme, 0.25 μM; pH, 10.0; temperature, 30˚C.

E.coli BL21(DE3) pET-32a-GDH

Amino acid sequence:
MLKVIQSPAKYLQGPDAAVLFGQYAKNLAESFFVIADDFVMKLAGEKVVNGLQSHDIRC
HAERFNGECSHAEINRLMAILQKQGCRGVVGIGGGKTLDTAKAIGYYQKLPVVVIPTIAST
DAPTSALSVIYTEAGEFEEYLIYPKNPDMVVMDTAIIAKAPVRLLVSGMGDALSTWFEAK
ACYDARATSMAGGQSTEAALSLARLCYDTLLAEGEKARLAAQAGVVTEALERIIEANTY
LSGIGFESSGLAAAHAIHNGFTILEECHHLYHGEKVAFGTLAQLVLQNSPMDEIETVLGFC
QRAGLPVTLAQMGVKEGIDEKIAAVAKATCAEGETIHNMPFAVTPESVHAAILTADLLGQQ
WLAR

Molecular weight: 54 kDa
